# Sustainable Zeolite–Silver Nanocomposites via Green Methods for Water Contaminant Mitigation and Modeling Approaches

**DOI:** 10.3390/nano14030258

**Published:** 2024-01-25

**Authors:** Álvaro de Jesús Ruíz-Baltazar, Simón Yobanny Reyes-López, Néstor Méndez-Lozano, Nahum Andrés Medellín-Castillo, Ramiro Pérez

**Affiliations:** 1 CONAHCYT-Centro de Física Aplicada y Tecnología Avanzada, Universidad Nacional Autónoma de México, Boulevard Juriquilla 3001, Santiago de Querétaro 76230, Mexico; 2 Laboratorio de Materiales Híbridos Nanoestructurados, Instituto de Ciencias Biomédicas, Departamento de Ciencias Químico-Biológicas, Universidad Autónoma de Ciudad Juárez, Anillo Envolvente del Pronaf y Estocolmo s/n, Zona Pronaf, Ciudad Juárez 32310, Mexico; simon.reyes@uacj.mx; 3 Campus Querétaro, Universidad del Valle de México, Blvd. Juriquilla No. 1000 A Del., Santa Rosa Jáuregui 76230, Mexico; nestor.mendez@uvmnet.edu; 4 Facultad de Ingeniería, Universidad Autónoma de San Luis Potosí, Av. Dr. Manuel Nava No. 8, Zona Universitaria, San Luis Potosí 78210, Mexico; 5 Instituto de Ciencias Físicas, Universidad Nacional Autónoma de México, Av. Universidad s/n, Col. Chamilpa, Cuernavaca 62210, Mexico; ramiro@icf.unam.mx

**Keywords:** sustainable adsorbent, green-synthesized nanoparticles, kinetic adsorption models, contaminant remediation, Zeolite–silver nanocomposites, copper removal

## Abstract

This study explores cutting-edge and sustainable green methodologies and technologies for the synthesis of functional nanomaterials, with a specific focus on the removal of water contaminants and the application of kinetic adsorption models. Our research adopts a conscientious approach to environmental stewardship by synergistically employing eco-friendly silver nanoparticles, synthesized using *Justicia spicigera* extract as a biogenic reducing agent, in conjunction with Mexican zeolite to enhance contaminant remediation, particularly targeting Cu^2+^ ions. Structural analysis, utilizing X-ray diffraction (XRD) and high-resolution scanning and transmission electron microscopy (TEM and SEM), yields crucial insights into nanocomposite structure and morphology. Rigorous linear and non-linear kinetic models, encompassing pseudo-first order, pseudo-second order, Freundlich, and Langmuir, are employed to elucidate the kinetics and equilibrium behaviors of adsorption. The results underscore the remarkable efficiency of the Zeolite–Ag composite in Cu^2+^ ion removal, surpassing traditional materials and achieving an impressive adsorption rate of 98% for Cu. Furthermore, the Zeolite–Ag composite exhibits maximum adsorption times of 480 min. In the computational analysis, an initial mechanism for Cu^2+^ adsorption on zeolites is identified. The process involves rapid adsorption onto the surface of the Zeolite–Ag NP composite, followed by a gradual diffusion of ions into the cavities within the zeolite structure. Upon reaching equilibrium, a substantial reduction in copper ion concentration in the solution signifies successful removal. This research represents a noteworthy stride in sustainable contaminant removal, aligning with eco-friendly practices and supporting the potential integration of this technology into environmental applications. Consequently, it presents a promising solution for eco-conscious contaminant remediation, emphasizing the utilization of green methodologies and sustainable technologies in the development of functional nanomaterials.

## 1. Introduction

Environmental pollution remains an urgent global concern, demanding innovative strategies aligned with sustainability principles and cleaner production practices. Among the critical environmental challenges, the contamination of water resources by heavy metals and other hazardous substances, such as organic dyes, pesticides, drugs, antibiotics, and phenols [1,2], stands as a transcendental threat to ecosystems and human well-being. In this context, the development of sustainable and eco-friendly methods for contaminant removal assumes paramount importance. Also in this context, it is crucial to highlight that the primary origins of heavy metals in the surroundings encompass electroplating, battery manufacturing, mining, pulp production, paint manufacturing, metal melting, pigment creation, ceramics, and related sectors [3,4,5]. Metals such as mercury (Hg), lead (Pb), nickel (Ni), zinc (Zn), cadmium (Cd), chromium (Cr), and Copper (Cu) are prevalent heavy metals, classified as perilous waste and acknowledged for their teratogenic and carcinogenic properties [6,7]. The pronounced contamination of aquatic ecosystems caused by heavy metal presence represents a crucial and pressing concern, underscoring the necessity to address this global challenge [8,9]. In this regard, various methodologies for water remediation have been reported, including ion exchange, electrochemical treatment, chemical precipitation, water dilution, adsorption, and sediment neutralization through biological and catalytic methods [10,11,12,13]. Among these, the adsorption technique has gained significant recognition for its eco-friendly, convenient, and highly efficient attributes. Therefore, one promising avenue for addressing this challenge involves the application of adsorption-based removal systems, known for their effectiveness and environmental compatibility. In recent years, researchers have increasingly explored the utilization of green-synthesized nanoparticles in tandem with naturally occurring zeolites to create high-performance adsorbent materials [14,15]. This approach combines the unique properties of both components to achieve enhanced contaminant removal while minimizing the ecological footprint of the process.

On the one hand, Zeolites are aluminosilicates with crystalline microporous structures, extensively utilized as catalysts, adsorbents, and ion exchangers [16,17,18,19,20]. They are classified as tectosilicate-type minerals and, due to their specific pore sizes, thermal stability, and high disponibility, the zeolites offer an excellent alternative for water remediation.

On the other hand, silver nanoparticles (AgNPs) have been the focus of considerable interest due to their unique adsorption capabilities, superior conductivity, chemical stability, and antimicrobial properties, which are all vital for efficient water treatment. Additionally, their antibacterial properties make them highly effective against a broad spectrum of microorganisms, including viruses, bacteria, and fungi. They are commercially applied in point-of-use water treatment as a post-treatment for filtrate water [21,22,23,24,25]. However, traditional AgNPs synthesis methods often entail the use of harsh chemicals and energy-intensive processes, presenting substantial environmental concerns. In contrast, green synthesis methods leverage biogenic reducing agents derived from natural sources, aligning seamlessly with the principles of cleaner production and sustainable chemistry.

Recent research involves utilizing various parts of plants like leaves, roots, flowers, fruits, and rhizomes as effective reducing agents for synthesizing AgNPs [26]. Plants such as guava, neem, aloe vera, Momordica charantia, Eclipta alba, lemon, hibiscus, Leptadenia reticulata, Clitoria ternatea, and tulasi have gained prominence in AgNP synthesis. Moreover, a considerable body of research focuses on investigating the antibacterial activity of these Ag nanoparticles [27]. An important method for green synthesis entails employing an extract from Justicia Spicigera as a reducing agent to produce silver nanoparticles (AgNPs). This method not only mitigates the environmental impact associated with nanoparticle synthesis but also harnesses the intrinsic potential of bioactive compounds for sustainable materials development. In parallel, the incorporation of AgNPs into naturally abundant zeolites, readily available in regions such as Mexico, offers a unique opportunity to engineer high-performance adsorbent materials. Zeolites possess well-defined porous structures and ion-exchange capabilities, rendering them ideal candidates for adsorption-based contaminant removal applications [28].

The primary objective of this study is to present a systematic investigation into the development and application of kinetic adsorption models for sustainable contaminant removal. We leverage the synergistic effects of green-synthesized AgNPs and Mexican zeolite to craft an efficient and eco-friendly adsorption system capable of removing hazardous contaminants from water resources. Likewise, in this work, we comprehensively address the synthesis, characterization, and experimental approach for contaminant elimination of the composite material. With respect to adsorption models, it is crucial to highlight that both first- and second-order models, linear or non-linear, are routinely employed in adsorption kinetics studies. These models consider factors such as external mass transfer, intraparticle diffusion, and adsorption at specific sites. Linear models can often adequately fit experimental data, particularly when the adsorbate concentration is low. However, non-linear models may offer a superior fit in instances where the adsorbate concentration is high. As such, a comprehensive evaluation of all factors influencing the Cu adsorption process is necessary to accurately decipher the specific aspects of the Cu adsorption phenomenon in the Zeolite–Ag composite. This allows for the establishment of a specific adsorption mechanism for this Zeolite, Ag, and Cu^2+^ system, as proposed in this work. The results presented in this study unequivocally demonstrate the superior efficacy of the composite in degrading Cu^2+^ ions, outperforming conventional materials. In essence, this research represents a fusion of sustainable synthesis approaches with readily available materials, presenting a promising avenue for addressing the pressing requirement for sustainable contaminant removal technologies. Our findings make a significant contribution to the expanding realm of knowledge on cleaner production practices, offering a new and eco-friendly approach to water purification and environmental remediation.

## 2. Materials and Methods

### 2.1. Zeolite Preparation

In this study, a Zeolite sourced from Oaxaca was utilized as the primary adsorbent material. The Zeolite underwent a series of preparatory steps to ensure its suitability for contaminant removal. Initially, the raw Zeolite was mechanically milled and sieved to achieve a particle size of −120 + 60 mesh (2 mm). To enhance its purity and remove impurities, a thorough washing treatment was conducted. The Zeolite was subjected to magnetic stirring at 700 rpm for 60 min. Subsequently, the material was carefully dried at a controlled temperature of 30 °C for 24 h.

### 2.2. Synthesis of Zeolite–Ag Composite

The synthesis of the Zeolite–Ag composite involved a two-stage process, commencing with the green synthesis of silver nanoparticles (AgNPs).

#### 2.2.1. Green Synthesis of Silver Nanoparticles

A total of 5.0 g of dried and milled *Justicia Spicigera* plant material was mixed with 30 mL of deionized water. This mixture was heated to 100 °C and maintained at this temperature for 20 min. Subsequently, the *Justicia Spicigera* solution was cooled to room temperature.

In parallel, a 50 mM solution of Silver Nitrate (AgNO_3_) of reagent grade with a purity of 99.98% was prepared in an aqueous medium. This chemical was sourced from Sigma-Aldrich (Spruce St, St. Louis, MO, USA, EE.UU). The solutions obtained from the previous steps were combined. After 1 h, a noticeable color change from dark brown to dark gray signified the successful synthesis of AgNPs.

#### 2.2.2. Homogeneous Nucleation of Ag Nanoparticles into Zeolite

Initially, 10 mg of Zeolite, which had been previously meshed to 200 mesh, washed, and dried, was diluted in 30 mL of deionized water. The Ag nanoparticles, as previously prepared, were introduced into the Zeolite solution. The mixture underwent ultrasonic stirring for 15 min to facilitate the incorporation process. The resulting material was then dried at 30 °C until powders of the Zeolite–Ag composite were obtained.

### 2.3. Adsorption Experiments

To evaluate the adsorption capacity of both the Zeolite and the Zeolite–Ag complex for Cu^2+^ ions, adsorbate (Cu^2+^) and adsorbent concentrations were set at 2.5 mg/L and 20 g/L, respectively. The adsorbate/adsorbent solutions were subjected to ultrasonication at 40 kHz and 100 W (pH = 7). At defined time intervals (30 min), aliquots of the solutions were extracted and measured using Absorption Atomic Spectroscopy (AAS). The measurements were taken until the pollutant concentration reached its minimum value.

### 2.4. Kinetic Adsorption Models

To comprehensively assess the adsorption behavior of Cu^2+^ ions by both the Zeolite and the Zeolite–Ag complex, we employed both linear and non-linear kinetic adsorption models. These models allowed us to gain a thorough understanding of the interaction mechanisms governing the adsorption process between the adsorbent materials and the heavy metal ions. This robust experimental methodology facilitated the systematic investigation of contaminant removal using our synthesized materials, offering valuable insights into their performance and efficiency in the context of cleaner production and environmental sustainability. Table 1 shows the kinetic adsorption models employed [12,29,30,31]:

The curve fitting of the experimental data to both linear and nonlinear theoretical models, as outlined in Table 1, was executed using the software “Origin 9.2”. This software employs the least-squares algorithm, a method also known as chi-square minimization, to derive the curve fit. The primary objective of this method is to minimize the deviations between the theoretical curve(s) and the experimental points. The algorithm accomplishes this by selecting parameters that yield the smallest deviations.

The least-squares method is defined as follows:

(1) $$\chi^{2}=\sum_{i=1}^{n}{\left[\frac{Y_{i}-f(x_{i}^{\prime },\hat{\beta })}{\sigma_{i}}\right]}^{2}$$

where *x_i_*′ is the row vector for the *i*th (*i* = 1, 2, …, *n*) observation.

Furthermore, the Levenberg–Marquardt (L-M) algorithm was employed to adjust the parameter values in the iterative procedure. The L-M algorithm is an iterative procedure that combines the Gauss–Newton method and the steepest descent method. It works well for most cases and has become the standard of nonlinear least square routines.

## 3. Results and Discussion

### 3.1. Materials Characterization 

#### 3.1.1. Scanning Electron Microscopy SEM

**Figure 1:**
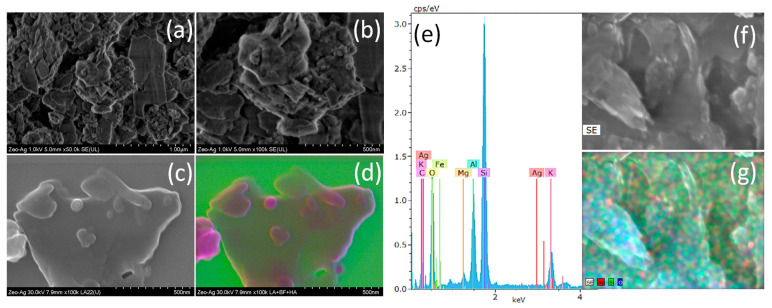
Scanning electron microscopy images depicting the structure of Zeolite and the distribution of silver nanoparticles. (**a**,**b**) Secondary electron micrographs at low magnifications, demonstrating the coexistence of Zeolite and silver nanoparticles. (**c**) Low-angle (LA) image of the Zeolite–Ag composite. (**d**) Composite representation with enhanced color for clarity. (**e**) Energy Dispersive X-Ray Spectroscopy (EDS) of Zeolite composite, (**f**) secondary electron image of the obtained zone of the EDS, and (**g**) EDS mapping of the Zeolite–Ag Composite.

Figure 1 showcases a series of scanning electron microscopy images that offer an in-depth visualization of the Zeolite structure and the distribution of silver nanoparticles within it. Figure 1a,b are secondary electron micrographs captured at low magnifications, which distinctly depict the coexistence of Zeolite and silver nanoparticles. These micrographs reveal the presence of silver nanoparticles with dimensions smaller than 50 nm on the Zeolite’s surface, signifying the successful implementation of the green synthesis method outlined in this study. Figure 1c presents a low-angle (LA) image that provides a unique perspective of the Zeolite–Ag composite, while Figure 1d is a composite representation with color enhancement to improve visual clarity. These images collectively demonstrate the successful integration of silver nanoparticles with Zeolite, substantiating our assertion that the green synthesis route we proposed was effective in achieving this result. Finally, Figure 1e–g represent an Energy Dispersive X-Ray Spectroscopy (EDS) of the Zeolite composite, a secondary electron image of the specific zone from which the EDS data were obtained, and an EDS mapping that provides a spatial distribution of elements within the Zeolite–Ag Composite, respectively. This EDS study confirms the concurrent presence of Zeolite and silver nanoparticles, further emphasizing the successful implementation of the green synthesis method outlined in this study.

#### 3.1.2. Transmission Electron Microscopy SEM

**Figure 2:**
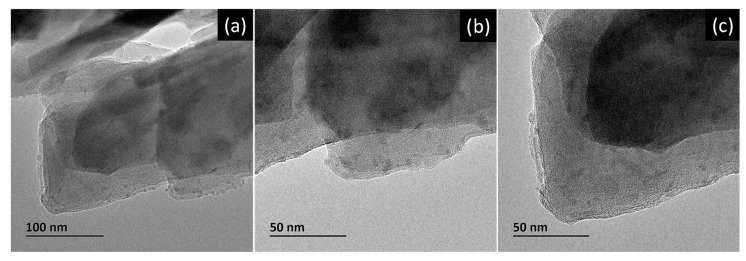
(**a**–**c**) Bright Field Transmission Electron Microscopy (BF-TEM) images of the Zeolite–Ag composite.

Figure 2a–c show bright field transmission electron microscopy (BF-TEM) images from the Zeolite–Ag composite, which illustrate the distribution of the Ag nanoparticles onto the Zeolite structure; examining these TEM images, it is possible to determine and corroborate the Ag nanoparticles’ formation and their particle size, which is approximately 5 nm. In this sense, it is important to mention that the resolution of the TEM allows for the resolution of the minimal particle size of the AgNPs; on the other hand, and in comparison, the SEM images did not reveal the smaller sizes of the AgNPs. Therefore, two particle size distributions presumably coexist: the first around 50 nm and the second approximately 5 nm. Additionally, it is essential to consider that the homogeneous porosity of the Zeolite and the pore size permit a selective interaction with different Ag particle sizes. Nevertheless, the behavior and Cu^2+^ adsorption efficiency is evaluated by taking a count of these factors, and using theoretical kinetic adsorption models, we can incorporate and describe as principal variables the initial and final concentrations of Cu^2+^ in the solution, such as the adoption time. The parameter derived from these models describes in detail the adoption of Cu^2+^ behavior.

#### 3.1.3. X-ray Diffraction Analysis

**Figure 3:**
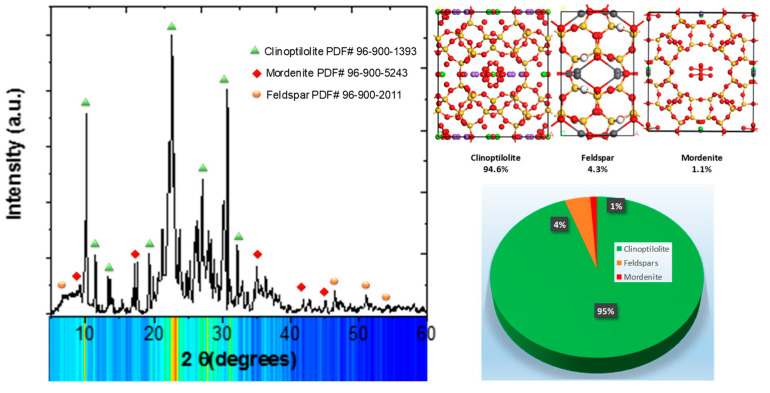
X-ray diffraction (XRD) pattern and quantification phase analysis of the natural Zeolite.

In Figure 3, we present the X-ray diffraction (XRD) pattern resulting from the treatment of natural Zeolite powders with deionized water. As illustrated in the figure, the XRD pattern distinctly showcases well-defined peaks corresponding to the characteristic clinoptilolite crystalline structure as a main phase. Concurrently, the residual peaks observed in the XRD pattern can be attributed to the coexistence of mordenite and feldspar phases within the sample.

The clinoptilolite possesses a monoclinic crystalline structure with the following lattice parameters: a = 17.65 Å, b = 17.92 Å, c = 7.403 Å, and β = 116.39°. This crystallographic arrangement corresponds to the C_2_/m space group (PDF# 96-900-1393). Additionally, the Mordenite phase exhibits an orthorhombic crystalline structure characterized by lattice parameters of a = 18.16 Å, b = 20.45 Å, and c = 7.54 Å. This crystal structure aligns with the Cmc21 space group and is indexed under entry PDF#96-900-5243. Conversely, the feldspar phase is distinguished by a monoclinic crystal lattice, featuring lattice parameters of a = 8.544 Å, b = 12.99 Å, c = 7.181 Å, and β = 116.16°. This particular crystallographic configuration is classified under the C2/m space group and is annotated as entry PDF#96-900-2011. In order to substantiate the observed results, a quantitative phase analysis was conducted. As illustrated in Figure 3, the analysis revealed a composition comprising a 94.3% weight of the clinoptilolite phase, 4.3% of feldspar, and 1.1% of the mordenite. These findings suggest that the adsorption properties inherent to the natural zeolite primarily emanate from the clinoptilolite structural component. Subsequent analyses included the modeling of the clinoptilolite structure to further elucidate its properties and potential applications.

### 3.2. Adsorption Study of Cu^2+^ by Zeolite and Zeolite–Ag

**Figure 4:**
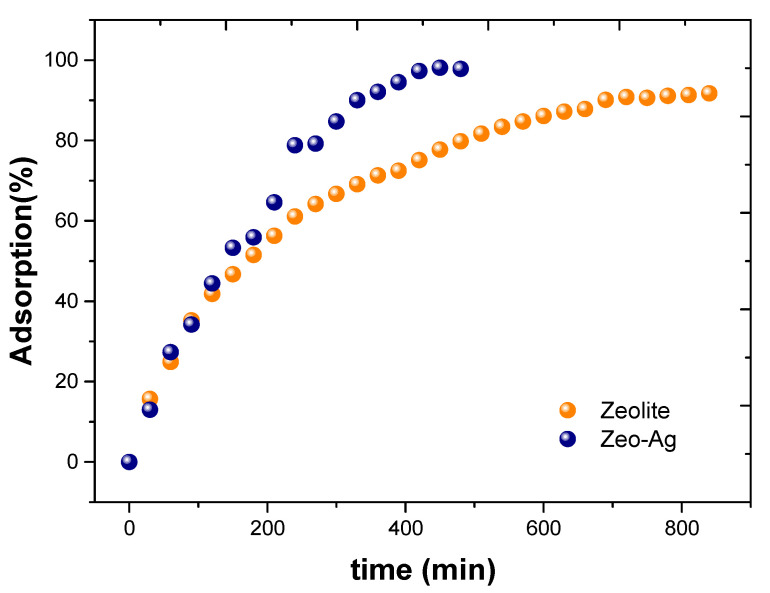
Experimental data of Cu^2+^ adsorption onto natural Zeolite and Zeolite–Ag composites.

Concerning the adsorption of Cu^2+^ onto the Zeolite and Zeolite–Ag composite materials, Figure 4 presents the experimental data obtained through Atomic Absorption Spectroscopy (AAS) measurements. Notably, we observed maximum adsorption times of 840 min for Zeolite and 480 min for the Zeolite–Ag composite, representing a significant reduction of 360 min in the latter case, which can be attributed to the presence of silver nanoparticles (AgNPs) on the Zeolite surface.

This observed phenomenon can be ascribed to the synergistic interaction between Zeolite and AgNPs. Firstly, this synergy reduces the Cu adsorption time. Secondly, it enhances the Cu adsorption percentage, reaching 91% for pristine Zeolite and 98% for the Zeolite–Ag composite. This behavior can be explained by the intrinsic ionic exchange capacity of Zeolite and the Van der Waals attraction forces associated with the negative charges of the AgNPs, as shown previously by the authors of reference [32]. In essence, this combined effect facilitates Cu adsorption, which is corroborated by the AAS measurements shown in Figure 4.

### 3.3. Kinetic Adsorption Models

#### 3.3.1. Linear Models Adsorption Models

**Figure 5:**
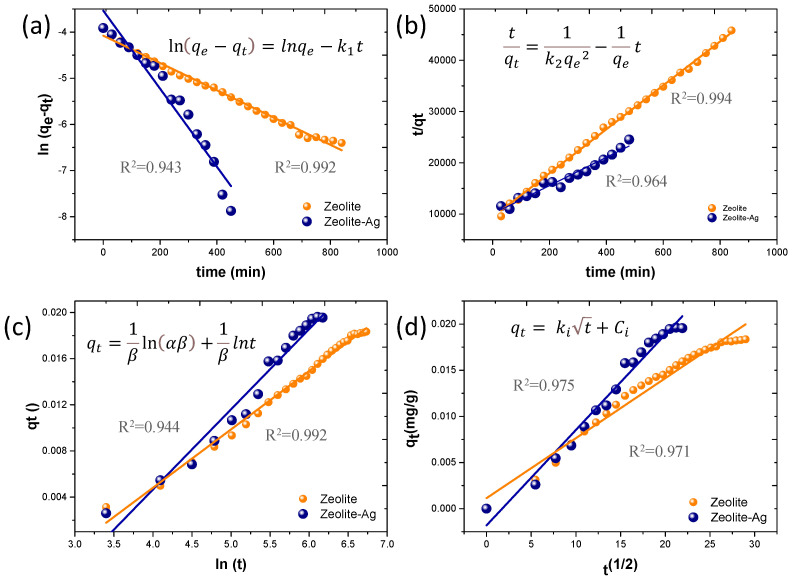
(**a**–**d**) Experimental data and modeling using pseudo-first-order, pseudo-second-order, Elovich, and Intraparticle Diffusion Models.

To determine the most suitable kinetic adsorption model for the experimental data from the Cu adsorption process, we evaluated the pseudo-first-order, pseudo-second-order, Elovich, and Intraparticle Diffusion Models, as depicted in Figure 5a–d. The equations corresponding to each kinetic adsorption model are included within their respective graphs. Impressively, the highest correlation coefficients (R^2^) were observed for the pseudo-second-order model. The detailed kinetic parameters are presented in Table 2.

In this context, the Ho kinetic adsorption model, also known as the pseudo-second-order model, provides the best fit for describing the Cu^2+^ adsorption mechanism by both Zeolite and the Zeolite–Ag composite. The pseudo-second-order model signifies a reversible second-order reaction, particularly relevant under low sorbate-to-sorbent ratios. Consequently, the Cu adsorption process is governed by two primary factors: ionic exchange within the Zeolite and Van der Waals attraction forces arising from the negative charges of the AgNPs.

#### 3.3.2. Non-Linear Adsorption Models

Non-linear adsorption models were employed in this study to compare their performance with linear models in the context of adsorption phenomena. Four distinct models were proposed, including two variations of the pseudo-first-order model and two non-linear models corresponding to the pseudo-second-order model presented in the experimental methodology [31,33,34,35]

**Figure 6:**
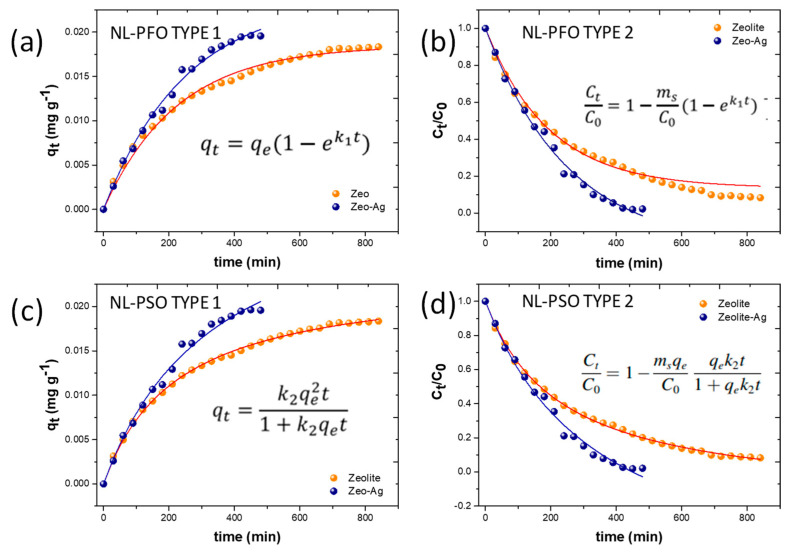
Non-linear forms of the pseudo-first-order (**a**,**b**) and pseudo-second-order (**c**,**d**) models.

In both cases, the parameters involved in these models serve to characterize the adsorption rates and the quantity of adsorbate and sorbate engaged in the process. Figure 6 illustrates the application of these non-linear adsorption models to the Zeolite and Zeolite–Ag composite.

Based on the calculated correlation factors, the non-linear models offer a superior description of the Cu adsorption mechanism on the Zeolite–Ag composite (Table 3) compared to the linear models presented earlier. It is noteworthy that the parameters used to derive the non-linear models, such as the initial and final absorbate concentrations, provide a highly precise depiction of the adsorption phenomena due to the more rigorous control of sorbate behavior.

In a broader context, both the linear and non-linear pseudo-second-order models exhibit superior precision in describing the adsorption process compared to the pseudo-first-order models. The nature of the pseudo-second-order models suggests a reversible second-order reaction at low sorbate-to-sorbent ratios. Consequently, the Cu adsorption process is governed by two primary phenomena: the ionic exchange within the Zeolite and the Van der Waals attractive forces associated with the negative charges of the Ag nanoparticles. Previous research has indicated that the typical exchange capacity of the mineral and the reactivity of Ag nanoparticles can synergistically influence the Cu^2+^ adsorption process [36,37,38]. Specifically, the polarization of the Zeolite in aqueous solutions and the electrostatic interactions of Ag nanoparticles contribute to the formation of negative potentials within the metal–oxygen bonds associated with the Zeolite [39,40].

**Figure 7:**
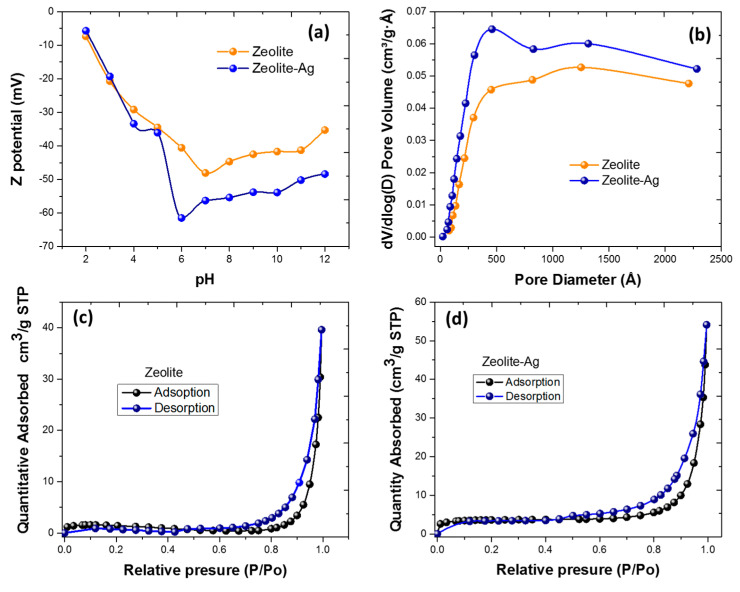
(**a**) Zeta potential vs. pH plot associated with the Zeolite and Zeolite–Ag composite, illustrating the net negative charge observed in both Zeolite and Zeolite–Ag samples. (**b**) Pore size distribution of the Zeolite and Zeolite–Ag samples. (**c**,**d**) N2 adsorption–desorption isotherms for both the Zeolite and Zeolite–Ag samples.

To elucidate and support these results, Figure 7a shows the graph of the Z potential vs pH, where a net negative charge of the Zeolite and the Zeolite–Ag compound can be observed. However, the Zeolite–Ag sample exhibits a higher negative Zeta potential, which could indicate that both the zeolite and silver nanoparticles contribute jointly to generating a more pronounced net negative charge, particularly at acidic pH levels. This can be attributed, presumably, to several factors among which could be the surface charge of silver nanoparticles, which can generate an affinity for adsorbing negatively charged species from the surrounding solution, causing an excess adsorption of negatively charged ions and resulting in a negative net charge at the Zeolite–Ag interface. Lastly, if both the Zeolite and silver nanoparticles exhibit acidic characteristics, their interaction results in a negative net charge. Acid–base reactions create negatively charged sites on the Zeolite, further accentuating the observed negative Zeta potential. This fact implies that the Zeolite–Ag composite exhibits a modified surface charge, suggesting an increased affinity and propensity for copper ion removal in relation to the undoped Zeolite. 

The Zeolite and Zeolite–Ag samples were characterized by N_2_ physisorption. The results, as depicted in Figure 7b, demonstrate that the Zeolite sample possesses a specific area of 4.6 m^2^/g, a pore volume of 0.02669 cm^3^/g, and an average pore diameter of 23.35 nm. These findings suggest a predominant presence of mesopores, according to the IUPAC classification.

In the case of the Zeolite–Ag Composite sample (Figure 7b), an enhancement in porosity is evident, as the specific area increased 2.7 times compared to the Zeolite sample. Specifically, the composite exhibits a specific area of 12.4 m^2^/g, a pore volume of 0.043882 cm^3^/g, and an average pore diameter of 14.2 nm. This substantial increase in specific area signifies improved porosity in the composite material.

Additionally, the N_2_ adsorption–desorption isotherms for both the Zeolite and Zeolite–Ag samples are presented in Figure 7c,d, respectively. In both cases, the isotherms conform to the type III isotherm characteristic of materials with low porosity. A type H3 loop hysteresis is observed, indicative of non-filling and condensation, contributing to the understanding of the materials’ porosity.

Moreover, the Cu^2+^ adsorption capacity is favorably influenced by the increased surface area of the Zeolite–Ag Composite. This result aligns with and reinforces the earlier presented findings, emphasizing the potential practical implications of our research.

This comprehensive analysis underscores the effectiveness of non-linear adsorption models, particularly the pseudo-second-order models, in elucidating the Cu^2+^ adsorption mechanism on the Zeolite–Ag composite, providing valuable insights into the complex interplay of adsorption processes and contributing to the understanding of adsorption phenomena in environmental remediation.

### 3.4. Non-Linear Langmuir and Freundlich Adsorptions Isotherms

According to the results obtained from non-linear PFO and PSO models, it is possible to affirm that the pseudo-second-order model describes in best form the adsorption of Cu^2+^ by the Zeolite and Zeolite–Ag complex.

**Figure 8:**
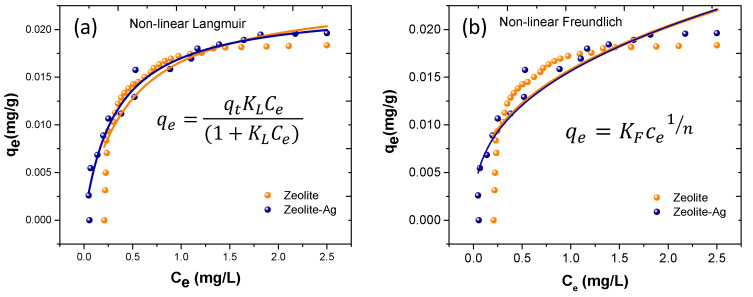
Non-linear models for (**a**) Langmuir and (**b**) Freundlich isotherms illustrating the adsorption of Cu^2+^ by Zeolite and Zeolite–Ag composite.

Nonetheless, a thorough investigation into the adsorption of Cu^2+^ utilizing Zeolites and Zeolite–Ag is crucial. In this regard, non-linear models for Langmuir and Freundlich isotherms were developed, as illustrated in Figure 8a,b, respectively. The objective was to accurately depict the uniformity of Cu^2+^ adsorption on the material surfaces. The Langmuir isotherm, a theoretical model portraying substance adsorption forming a monolayer on a solid surface, was examined. The proposed model offers a more precise description, considering saturation effects and cooperativity among adsorbed molecules. The non-linear Langmuir equation defines the relationship between the quantity of substance adsorbed on the surface (*q_e_*) and the concentration in solution (*C_e_*). This model presupposes a finite number of adsorption sites on the surface. Furthermore, a non-linear model for the Freundlich equation was developed, a more general model than Langmuir that is capable of representing multilayer adsorption on the solid surface. The Freundlich model is characterized by its adsorption exponent (*n*), which signifies surface heterogeneity and adsorption intensity. The non-linear Langmuir and Freundlich isotherms are mathematically governed by the subsequent expressions.

(2) $$q_{e}=\frac{q_{t}K_{L}C_{e}}{(1+K_{L}C_{e})\;}$$

(3) qe=KFCe1n

Based on the results obtained for the rate constants *K_L_* and *K_f_*, as well as the correlation factors R^2^, it is affirmed that the non-linear models of PFO and PSO exhibit notably high correlation factors, thereby providing a more accurate description of the Cu^2+^ adsorption phenomenon. Notably, the R^2^ values for the non-linear Langmuir and Freundlich isotherms were 0.96 and 0.90, respectively, for the Zeolite–Ag sample. In contrast, for the pristine Zeolite, these values were relatively lower (0.81 and 0.68). However, this outcome reveals significant information regarding monolayer formation on the surface of the Zeolite and Zeolite–Ag samples. In essence, the surface of the Zeolite and Zeolite–Ag samples behaves heterogeneously, resulting in the adsorption of Cu^2+^ ions described by a physisorption phenomenon, presumably due to electrostatic forces’ interactions between the adsorbent and adsorbate. Furthermore, it can be asserted that the incorporation of Ag nanoparticles enables the creation of a more homogeneous adsorption surface on Zeolite. This could be associated with the enhanced stability of electrostatic charges and an increased number of exchange sites within the Zeolite–Ag sample. Another crucial factor that could potentially influence the adsorption efficiency of the Zeolite–Ag composite is the presence of structural defects in the system, specifically at the interface between the dopant metals and the Zeolite. These defects have been reported to play a critical role in influencing the adsorption performance [41].

**Figure 9:**
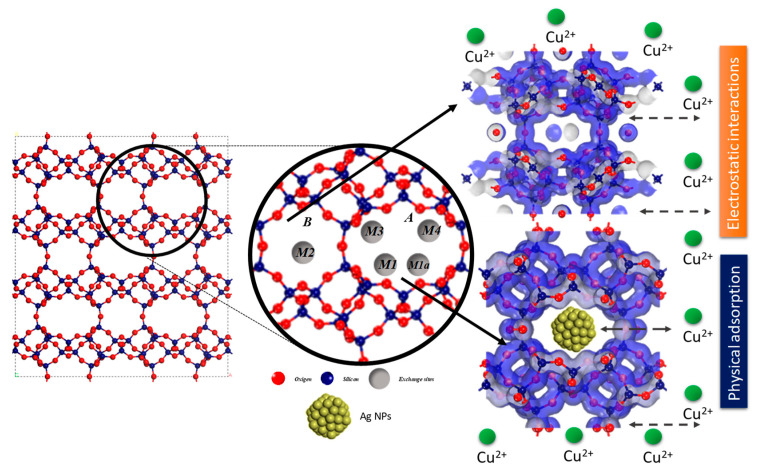
Structure of clinoptilolite-type Zeolite viewed along the c-vector, showcasing primary channels A and B, along with extra framework cation sites and their nucleophilic and electrophilic sites, and their interaction with adsorbed Cu^2+^ ions.

In order to comprehensively describe and discuss the obtained results, Figure 9 presents the structure of clinoptilolite-type Zeolite viewed along the c-vector. This view showcases the primary channels A and B, along with the extra framework cation sites and their nucleophilic and electrophilic positions. It also illustrates their interaction with adsorbed Cu^2+^ ions. In this context, the figure delineates the structural characteristics of clinoptilolite, which adopts a monoclinic crystal structure, belonging to the C_2_/m space group. Its structural framework consists of four- and five-membered rings, while the channel system is defined by eight- and ten-membered rings [42,43]. Within this structure, three principal cationic sites exist, hosting alkali and alkaline–earth cations. Two of these sites, labeled as M1 and M3, are located within channel A and are coordinated with oxygen atoms from the eight-membered lateral ring. The third crucial site, M2, is positioned within channel B. Additionally, a fourth site, M4, with significantly lower occupancy, was identified for hydrated cations of small size at the center of channel A. Notably, there is a distinction between sites M1 and M3, where the latter is more confined within the eight-membered lateral ring and less exposed to the channel. Sites M1 and M1a are shifted towards the center of the channel, with M1a is weakly coordinated by atoms from the lateral ring, albeit closer to the T1-O4-T2 and T1-O6-T5 bonds of the 10-membered ring within the channel.

## 4. Conclusions

In summary, the present research describes and analyzes the removal of copper ions (Cu^2+^) carried out by natural Zeolite and the Zeolite–Ag composite, which represents promising applications in water purification and environmental remediation. As a discussion, it can be stated that the mechanism of Cu^2+^ adsorption is mainly described by a physisorption process and electrostatic interactions between the Zeolite doped with Ag nanoparticles and the Cu^2+^ ions. Conversely, the integration of silver nanoparticles (AgNPs) into the Zeolite structure significantly amplifies its capacity for copper ion removal. AgNPs serve as highly efficient adsorption sites for copper ions, benefiting from their expansive surface area and strong affinity for heavy metal ions like Cu^2+^. This synergistic effect combines the ion-exchange capacity of Zeolite with the adsorption prowess of AgNPs. Moreover, the efficacy of copper ion removal by Zeolite–AgNPs stems from the selectivity and affinity of the Zeolite framework and AgNPs for Cu^2+^ ions. Copper ions demonstrate a noteworthy affinity for both the negatively charged sites within the Zeolite structure and the AgNPs, driven by electrostatic interactions and coordination chemistry. Consequently, Cu^2+^ ions preferentially adsorb onto the surface of Zeolite–AgNPs. The removal of copper ions by Zeolite–AgNPs follows distinct kinetic and equilibrium processes. In conclusion, the effective removal of copper ions by Zeolite–Ag nanoparticles is a complex interplay of ion-exchange processes, surface interactions, and the unique properties of both the Zeolite framework and the incorporated AgNPs. This approach offers a promising avenue for mitigating copper ion contamination in water and environmental systems, addressing a critical issue in water quality management and pollution control.

## Figures and Tables

**Table 1:** Kinetic adsorption models—linear and non-linear models.

Linear Models		Non-Linear Models	
Model	Equation	Model	Equation
Pseudo-First-Order (PFO)	$\ln\left(q_{e}-q_{t}\right)=ln{\;q}_{e}-kt$	Pseudo-First Order Type 1	$q_{t}=q_{e}(1-e^{k_{1}t})$
Pseudo-Second-Order (PSO)	$\frac{t}{q_{t}}=\frac{1}{k_{2}q_{e}^{2}}-\frac{1}{q_{e}}t$	Pseudo-First Order Type 2	$\frac{C_{t}}{C_{0}}=1-\frac{m_{s}}{C_{0}}(1-e^{k_{1}t})$
Elovich	$q_{t}=\frac{1}{\beta }\ln\left(\alpha \beta \right)+\frac{1}{\beta }t$	Pseudo-Second Order Type 1	$q_{t}=\frac{k_{2}q_{e}^{2}t}{1+k_{2}q_{e}t}$
Intraparticle Diffusion	$q_{t}=k_{i}\sqrt{t}+C_{i}$	Pseudo-Second Order Type 2	$\frac{C_{t}}{C_{0}}=1-\frac{m_{s}q_{e}}{C_{0}}\frac{q_{e}k_{2}t}{1+q_{e}k_{2}t}$

**Table 2:** Kinetic parameters obtained from linear models in the adsorption process (pseudo-first-order, pseudo-second-order, Elovich, and Intraparticle Diffusion Models).

Linear Models								
SAMPLE	PFO (Linear)		PSO (Linear) K_2_ (g/mgmin); q_e_(mg/g)		Elovich		Intraparticle Diffusion	
Zeolite	K_1_	0.0069	K_2_	0.1951	α	1715.125	Ci	0.00113
			q_e_	5.45 × 10^−4^	β	−337.838	Ki	0.00065003
	R^2^	0.9922	R^2^	0.994	R^2^	0.992	R^2^	0.971
Zeolite–Ag	K_1_	0.0197	K_2_	0.0758	α	536.699	Ci	−0.002
			q_e_	0.0013	β	−118.343	Ki	0.001
	R^2^	0.9437	R^2^	0.9641	R^2^	0.944	R^2^	0.975

**Table 3:** Kinetic parameters and correlation factors calculated for the non-linear forms of the pseudo-first-order and pseudo-second-order models.

MODEL	SAMPLE	TYPE 1		TYPE 2	
**NL Pseudo-First-Order**	**Zeolite**	**k_1_**	**0.01848**	**k_1_**	0.005158
		qe	0.00446	qe	0.05838
		R^2^	0.9923	m_s_	21.7614
		-	-	C_o_	1.4686
		-	-	R^2^	0.9987
	Zeolite-Ag	k_1_	0.02381	k_1_	0.0039
		qe	0.00399	qe	0.05953
		R^2^	0.9933	m_s_	22.1913
		-	-	C_o_	1.1096
		-	-	R^2^	0.9973
**NL Pseudo-Second-Order**	Zeolite	k_1_	0.1894	k_2_	0.0249
		qe	0.02348	qe	0.1786
		R^2^	0.9981	m_s_	19.7481
		-	-	C_o_	3.0042
		-	-	R^2^	0.9986
	Zeolite-Ag	k_1_	0.0829	k_2_	0.01343
		qe	0.03523	qe	0.21746
		R^2^	0.9985	m_s_	19.9989
		-	-	C_o_	2.4678
		-	-	R^2^	0.9965

## Data Availability

The data presented in this study are available on request from the corresponding author.
